# A Case of Pancreatic Neuroendocrine Tumor Growing Intraductal Extension toward the Main Pancreatic Duct Complicated by Thrombocytopenia: Diagnostic Challenges and Management Strategy

**DOI:** 10.1002/deo2.70241

**Published:** 2025-11-03

**Authors:** Koichi Soga, Kazuma Sakakibara, Yuki Soma, Manayu Shiina, Mayumi Yamaguchi, Masaru Kuwada, Ryosaku Shirahashi, Ikuhiro Kobori, Shinichi Ban, Masaya Tamano

**Affiliations:** ^1^ Department of Gastroenterology Dokkyo Medical University Saitama Medical Center Saitama Japan; ^2^ Department of Pathology Dokkyo Medical University Saitama Medical Center Saitama Japan

**Keywords:** main pancreatic duct, myelodysplastic syndrome, pancreatic neuroendocrine tumor, serial pancreatic juice aspiration cytologic examination, serotonin

## Abstract

We present a rare and diagnostically challenging case of a pancreatic neuroendocrine tumor (pNET) with intraductal growth into the main pancreatic duct (MPD), complicated by severe thrombocytopenia due to myelodysplastic syndrome. A 37‐year‐old male presented with thrombocytopenia. Abdominal imaging revealed an 11‐mm hypervascular lesion obstructing the MPD in the pancreatic body. The initial serial pancreatic juice aspiration cytological examination (SPACE) demonstrated Class II cytology. Eight months later, the second SPACE revealed Class V cytology. Pancreaticoduodenectomy confirmed pNET G2 with clear intraductal extension. In the postoperative specimen, a portion of the tumor was exposed within the MPD, suggesting Class V cytology. pNETs with intraductal extension (I‐pNETs) are rare, as pNETs typically exhibit expansive encapsulated growth. SPACE may be a valuable diagnostic alternative for patients with thrombocytopenia, although its accuracy may depend on factors such as capsular integrity and ductal communication. Tailored diagnostic strategies that balance invasiveness and safety are essential for managing pancreatic tumors in patients with hematological fragility. This case highlights the importance of considering I‐pNET in the differential diagnosis of MPD‐occupying lesions.

**Abbreviations**
CT: computed tomography
ERCP: endoscopic retrograde cholangiopancreatography
EUS‐FNA: endoscopic ultrasound‐guided fine‐needle aspiration
I‐pNETs: pNETs with intraductal extension
MDS: myelodysplastic syndrome
MPD: main pancreatic duct
PDAC: pancreatic ductal adenocarcinoma
pNET: pancreatic neuroendocrine tumor
serotonin‐pNETs: serotonin‐producing pNETs
SPACE: serial pancreatic juice aspiration cytological examination

## 1 Introduction

Pancreatic neuroendocrine tumors (pNETs) are uncommon, and those involving the pancreatic duct are rarer (<2% of all pNETs) [1]. pNETs typically exhibit expansile growth with round to oval tumor cells forming a clear border with the surrounding pancreatic parenchyma. Serotonin‐producing pNETs (serotonin‐pNETs) are rare but clinically important. Their marked desmoplastic reaction can cause main pancreatic duct (MPD) obstruction even in small tumors. Such cases highlight the need to consider serotonin‐pNETs in the differential diagnosis of MPD‐occupying lesions [2]. Here, we present a rare case of an intraductal extension pNET (I‐pNET) with positive serotonin staining complicated by thrombocytopenia due to myelodysplastic syndrome (MDS).

## 2 Case Report

**FIGURE 1:**
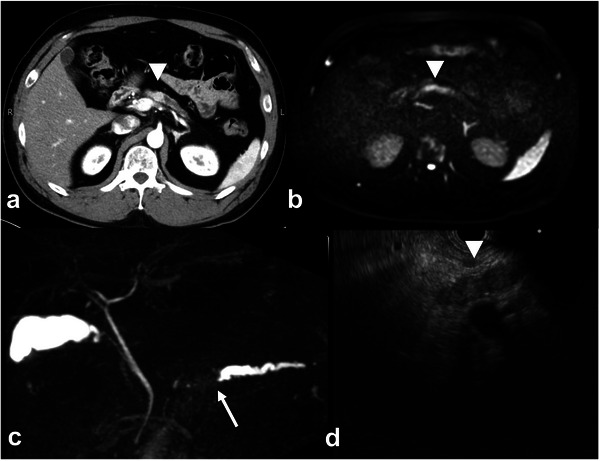
Pancreatic neuroendocrine tumor with intraductal growth into the main pancreatic duct at initial examination. Abdominal CT images showing a well‐enhanced mass in the pancreatic body during the arterial dominant phase (a). The mass protrudes toward the main pancreatic duct (MPD) of the pancreatic body, resulting in downstream dilation. On magnetic resonance imaging (MRI), the pancreatic neuroendocrine tumor (pNET) occupying the MPD revealed high signal intensity on diffusion‐weighted images (b). Magnetic resonance cholangiopancreatography demonstrates a dilated MPD (arrow) and a space‐occupying lesion within the pancreatic body with significant upstream dilatation of the duct in the body and tail of the pancreas, suggesting obstruction due to pNET (c). Endoscopic ultrasonography revealed a tumor filling the MPD within the pancreatic body. However, EUS could not determine whether the tumor originated within the MPD or from the pancreatic parenchyma (d).

A 37‐year‐old Japanese man presented to the hematology department with thrombocytopenia and macrocytic anemia. Screening computed tomography (CT) revealed a hypervascular mass in the pancreatic body adjacent to the MPD, which appeared to protrude into and obstruct the ductal lumen. The lesion measured 11 mm and caused upstream MPD dilation. Contrast‐enhanced CT revealed a well‐defined and homogeneous enhancement lesion in the early phase, with persistent enhancement in the later phases. MPD in the tail was dilated to 6 mm; atrophy of the pancreatic parenchyma without distant metastases was observed (Figure 1 and Video S1).

Because the lesion was within the MPD, the risk of duct injury with pancreatic juice leakage and bleeding associated with endoscopic ultrasound‐guided fine‐needle aspiration (EUS‐FNA) was a concern, especially given the low platelet count (15,000/µL). Therefore, after confirming platelet transfusion response, we performed endoscopic retrograde cholangiopancreatography (ERCP) and serial pancreatic juice aspiration cytological examination (SPACE). SPACE revealed Class II cytology, insufficient for a definitive diagnosis.

**FIGURE 2:**
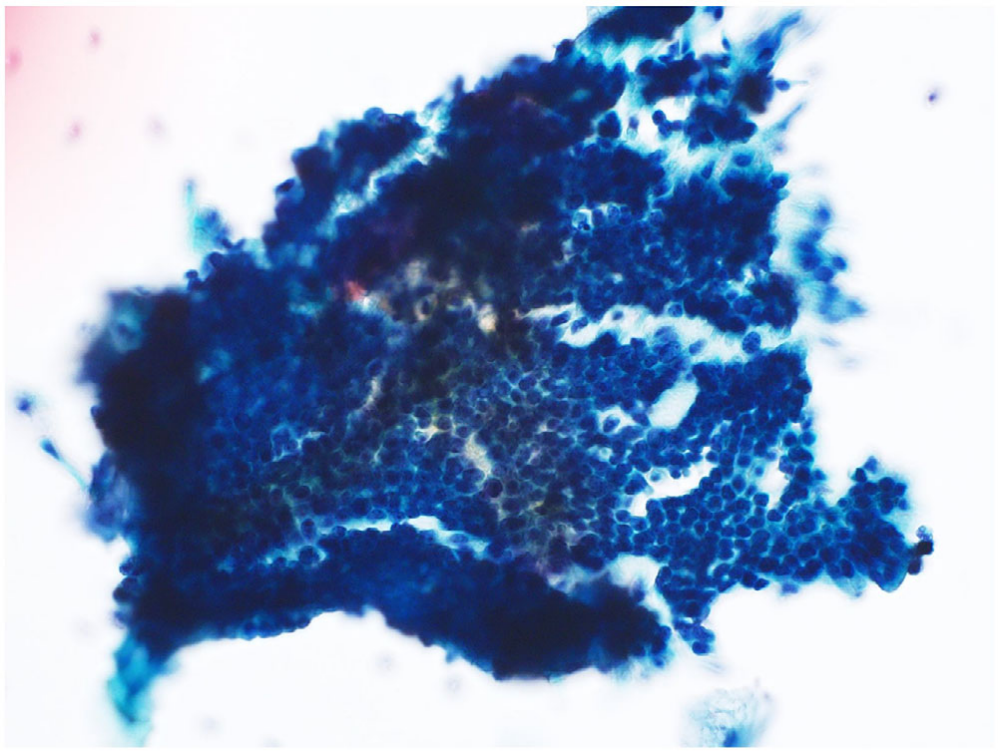
Cytological findings during the second endoscopic retrograde cholangiopancreatography with the selective pancreatic juice aspiration cytological examination (SPACE) procedure. Cytology revealed numerous clusters of atypical cells with nuclear enlargement, irregular architecture, increased chromatin, and prominent nucleoli, consistent with Class V.

Although resection would have been reasonable upon initial tumor detection, the patient presented with profound thrombocytopenia. Therefore, initial conservative management was selected, and a detailed hematological workup eventually led to MDS diagnosis. Positron emission tomography‐CT demonstrated focal fluorodeoxyglucose uptake, initially raising concern for bone metastasis; however, the absence of lytic or sclerotic lesions and the patient's known MDS supported a diagnosis of ineffective hematopoiesis and compensatory marrow hyperplasia rather than metastasis (Figure S1). Due to compliance issues, a second ERCP and SPACE were performed 8 months after the first. Cytology revealed numerous clusters of atypical cells with nuclear enlargement, irregular architecture, increased chromatin, and prominent nucleoli, consistent with Class V (Figure 2). Thus, pancreaticoduodenectomy was performed 10 months later, with perioperative platelet transfusion.

**FIGURE 3:**
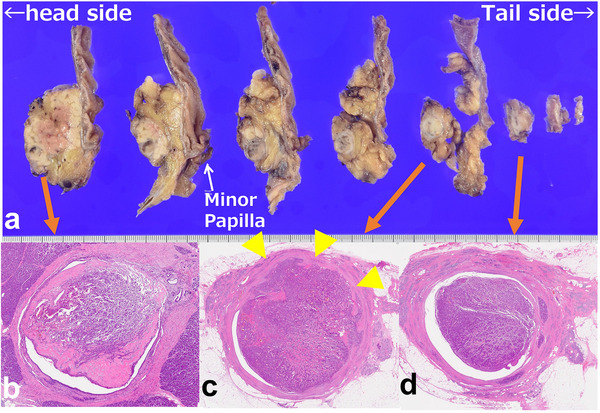
Histopathological findings of the resected pancreatic neuroendocrine tumor. Macroscopic appearance of resected tumor. Gross examination of the resected specimen revealed a solid, gray‐white, round mass extending from the pancreatic body, with focal hemorrhage (a). Microscopic findings. Serial histological sections from the head side to the tail side (b–d) demonstrated a well‐circumscribed, fibrously encapsulated tumor. Section (c), considered the site of tumor origin, showed the lesion protruding into the main pancreatic duct from the pancreatic parenchyma, with focal thinning of the capsule and exposure of tumor cells to the ductal lumen (arrowheads). Section (d) also demonstrated the tumor protruding into the main pancreatic duct, consistent with further intraductal extension. (Hematoxylin–eosin staining, original magnification ×40).

**FIGURE 4:**
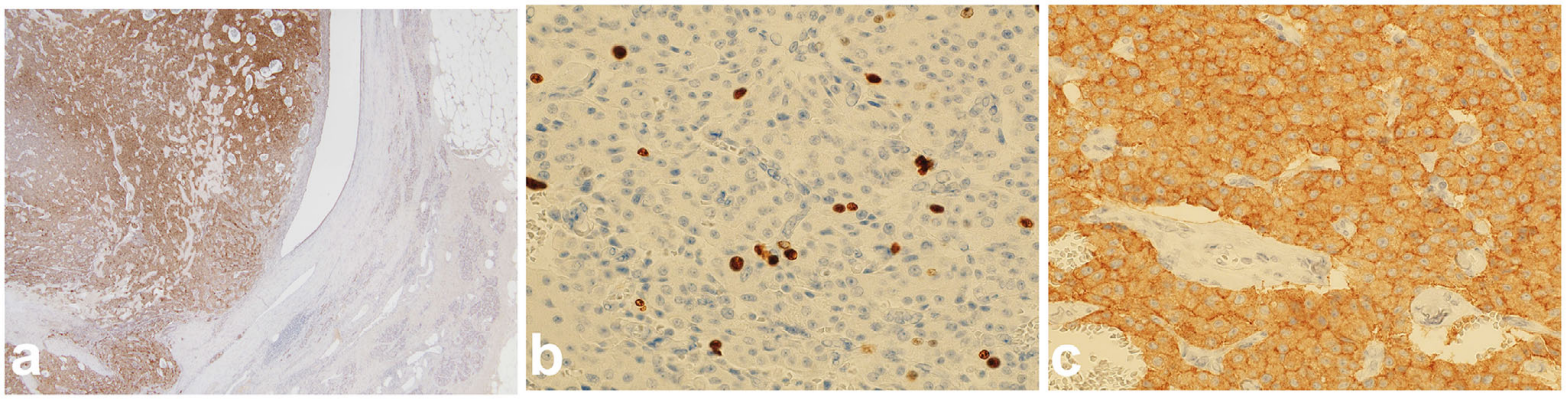
Immunohistopathological findings of the resected pancreatic neuroendocrine tumor. Immunohistochemical staining (×200) for serotonin in a pancreatic neuroendocrine tumor. The serotonin‐positive pancreatic neuroendocrine tumor occupied the main pancreatic duct. Staining revealed that the tumor originated from the pancreatic parenchyma and protruded into the ductal lumen (a). Ki‐67 immunohistochemical staining (×400) with a proliferation index of approximately 4.9% (b). Synaptophysin staining (×400) was diffusely positive in tumor cells (c).

Histologically, gross examination of the resected specimen revealed a solid, gray‐white, round mass extending from the pancreatic head to the body, with focal hemorrhage. The tumor appeared to originate near the MPD wall, protrude toward the dilated MPD, and compress the MPD epithelium, with some areas directly exposed to the lumen. Tumor growth was observed close to the dilated branch ducts of the pancreas, suggesting that the pNET originated from the pancreatic parenchyma with extensive intraductal involvement (Figure 3, Figures S2, and S3). Immunohistochemical staining was positive for chromogranin A, serotonin, and synaptophysin and negative for trypsin and alpha‐fetoprotein, with β‐catenin showing membranous positivity. The Ki‐67 labeling index was 4.9%; thus, pNET G2 was diagnosed (Figure 4). Lymph node metastases were absent. One year postoperatively, no recurrence has occurred. The patient is under strict follow‐up, with ongoing MDS treatment.

## 3 Discussion

This case is diagnostically challenging owing to a hypervascular tumor in the pancreatic body and profound thrombocytopenia secondary to MDS. The final diagnosis, serotonin‐pNETs protrude toward dilated MPD, was established after two SPACE sessions, with the second procedure confirming Class V cytology.

pNETs are typically well‐differentiated hypervascular neoplasms originating from Langerhans islet cells that exhibit tightly packed, uniform neuroendocrine cells with minimal stroma, often forming medullary patterns. They grow slowly and are encapsulated by a fibrous capsule separating them from the surrounding pancreatic parenchyma [3].

The histopathological characteristics of pNETs are radiologically apparent. pNETs typically appear as solid lesions with clear boundaries, mild internal heterogeneity, and rich capillary networks, producing strong homogeneous enhancement in the arterial phase of contrast‐enhanced CT. On magnetic resonance imaging, they exhibit a high signal intensity on T2‐weighted images [4] owing to their cellular density and low stromal content. These characteristics contrast the hypovascular and hypointense features of pancreatic ductal adenocarcinoma (PDAC), facilitating differential diagnosis. Given their rarity, I‐pNETs are often misinterpreted as intraductal papillary neoplasms or PDAC.

Pancreatic tumors with MPD involvement are characterized as expansile solid tumors and intraductal neoplasms [5]. A comparative summary of the radiological features of MPD‐occupying lesions is presented in Table S1. These features are valuable for preoperative differential diagnosis, although histological confirmation remains essential.

In our case, the pNET demonstrated marked intraductal extension with partial MPD obstruction. This pathogenesis suggests three causative factors: pancreatic duct compression, tumor extension into the duct, and duct constriction by a tumor with a fibrous stroma. The tumor originated near the MPD and expanded early into the ductal lumen (Figure 3). An intraductal extension may have occurred owing to local capsule rupture or mechanical pressure. The intraductal portion was more radiologically prominent than the parenchymal lesion, which may reflect a lower tissue pressure within the ductal system, allowing preferential growth into the duct. Histologically, serotonin‐pNETs typically exhibit a trabecular growth pattern and dense desmoplastic stroma. This fibrotic response is clinically relevant, as it may lead to pancreatic duct obstruction [4].

SPACE is useful in diagnosing PDAC in situ [6]. The cytological discrepancy between the two SPACE procedures may be explained by the histological features of pNETs. The first SPACE yielded Class II cytology, whereas the second revealed Class V cells, which initially suggested high‐grade adenocarcinoma; however, the final diagnosis was pNET. pNETs often possess a fibrous capsule that can limit cellular shedding into the pancreatic juice, which is especially pronounced in serotonin‐NETs characterized by dense peritumoral fibrosis. The first SPACE may have sampled an encapsulated portion, whereas the second may have sampled from a site of capsular disruption at the point of intraductal extension. Erosion may have occurred from abrasion of the pancreatic duct epithelium during insertion of the drainage tube, improving cytological yield. Furthermore, the displacement force between the tumor protruding like a polyp within MPD and the pancreatic parenchyma also influences the tumor's exposure within MPD. Therefore, the diagnostic yield of SPACE for pNETs depends on tumor exposure to the ductal lumen. This case is instructive in terms of the tumors occupying the pancreatic duct and their diagnostic process and underscores the potential and limitations of SPACE for I‐pNETs. Although SPACE is non‐invasive and safe for patients with bleeding risk, its diagnostic yield depends on anatomical factors. Furthermore, its diagnostic yield in pNETs is limited compared with that in PDAC, owing to the frequent encapsulation of pNETs. If the tumor predominantly involved the MPD, it would be pertinent to discuss the potential risk of pancreatic juice leakage associated with EUS‐FNA, as this complication may have important clinical implications in such cases.

The diagnostic approach was further complicated by severe thrombocytopenia due to MDS. Invasive procedures such as EUS‐FNA or ERCP with sphincterotomy pose significant bleeding risks [7]. A meta‐analysis reported a significantly higher bleeding risk after endoscopic procedures in patients with platelet counts <50,000/µL. Bleeding risk is 3‐fold higher in patients with platelet counts <25,000/µL. Thus, caution should be exercised when performing endoscopic interventions in such patients [8]. We opted for a low‐risk cytological approach using SPACE, accompanied by platelet transfusion. Platelet transfusion prevents bleeding but presents potential complications [9], particularly relevant in MDS; patients may require repeated transfusions, potentially leading to transfusion refractoriness. Thus, the decision to minimize procedural invasiveness while maintaining diagnostic accuracy was critical in this case.

We diagnosed I‐pNET with serotonin‐positive tumors in a patient with severe thrombocytopenia due to MDS. I‐pNET was diagnosed after two SPACE sessions; the second procedure confirmed Class V cytology. I‐pNETs should be included in the differential diagnosis of MPD, particularly when hypervascular intraductal pancreatic lesions are present. The tumor morphology renders diagnosis challenging based on pancreatic fluid or brush abrasion cytology alone. Thus, procedural risks should be carefully considered. In such complex settings, SPACE is a valuable diagnostic tool despite limitations.

## Author Contributions

Dr. Shiina contributed to reviewing and editing.

## Funding

The authors have nothing to report.

## Conflicts of Interest

The authors declare no conflicts of interest.

## Ethics Statement

The authors have nothing to report.

## Consent

Written consent for publication was obtained from the patient.

## Clinical Trial Registration

N/A.

## Supporting information




**FIGURE S1 Positron emission tomography‐computed tomography**. Coronal fused positron emission tomography‐computed tomography (PET‐CT) images were obtained using fluorodeoxyglucose (FDG). Volume control image showing focal FDG uptake, showing localization to the bone marrow (a). The delayed‐phase maximum intensity projection image highlights increased uptake in the bone marrow (b). PET‐CT with FDG uptake images in the bone marrow, raising the possibility of bone metastasis, although the findings were atypical for pancreatic cancer; however, the absence of lytic or sclerotic lesions and the patient's known MDS supported a diagnosis of ineffective hematopoiesis and compensatory marrow hyperplasia rather than metastasis.

**FIGURE S2 Pancreatic neuroendocrine tumor protruding into the main pancreatic duct and tumor cells exposed within the ductal lumen**. The pain pancreatic duct lumen (asterisk) and magnified view (box) demonstrate epithelial denudation of the duct wall, with tumor cells (arrow) directly exposed to the ductal lumen.

**FIGURE S3 Schematic representation of a serotonin‐positive pancreatic neuroendocrine tumor arising from the margin of the main pancreatic duct and protruding into the ductal lumen**. Tumor enlargement, mechanical stress, and shear forces between the tumor and the pancreatic parenchyma eventually caused disruption of the fibrous capsule, resulting in exfoliation of tumor cells into the main pancreatic duct. Cytological evaluation was performed using an endoscopic nasobiliary drainage (ENBD) tube. During the first SPACE examination, only class II cytology was obtained, with no definitive tumor cells detected. Although imaging findings showed no apparent differences between the first and second examinations, the second SPACE examination yielded class V cytology. This discrepancy was considered to reflect the progression of capsular rupture due to tumor growth, which facilitated the detachment and shedding of tumor cells into the ductal lumen, allowing their recovery through the ENBD tube. The yellow line delineates the extent of the tumor.

**TABLE S1** Differential diagnosis of main pancreatic duct (MPD)–occupying tumors.

**VIDEO S1** Images of a pancreatic neuroendocrine tumor (pNET) protruding into the main pancreatic duct (MPD) at the initial examination in this case. White arrowheads indicate points of protrusion.
