# Supplementary figures and images for: A Case of Pancreatic Neuroendocrine Tumor Growing Intraductal Extension toward the Main Pancreatic Duct Complicated by Thrombocytopenia: Diagnostic Challenges and Management Strategy
Source: DEN Open. 2025 Nov 3;6(1):e70241. doi: 10.1002/deo2.70241 (PMC12582909; doi:10.1002/deo2.70241)

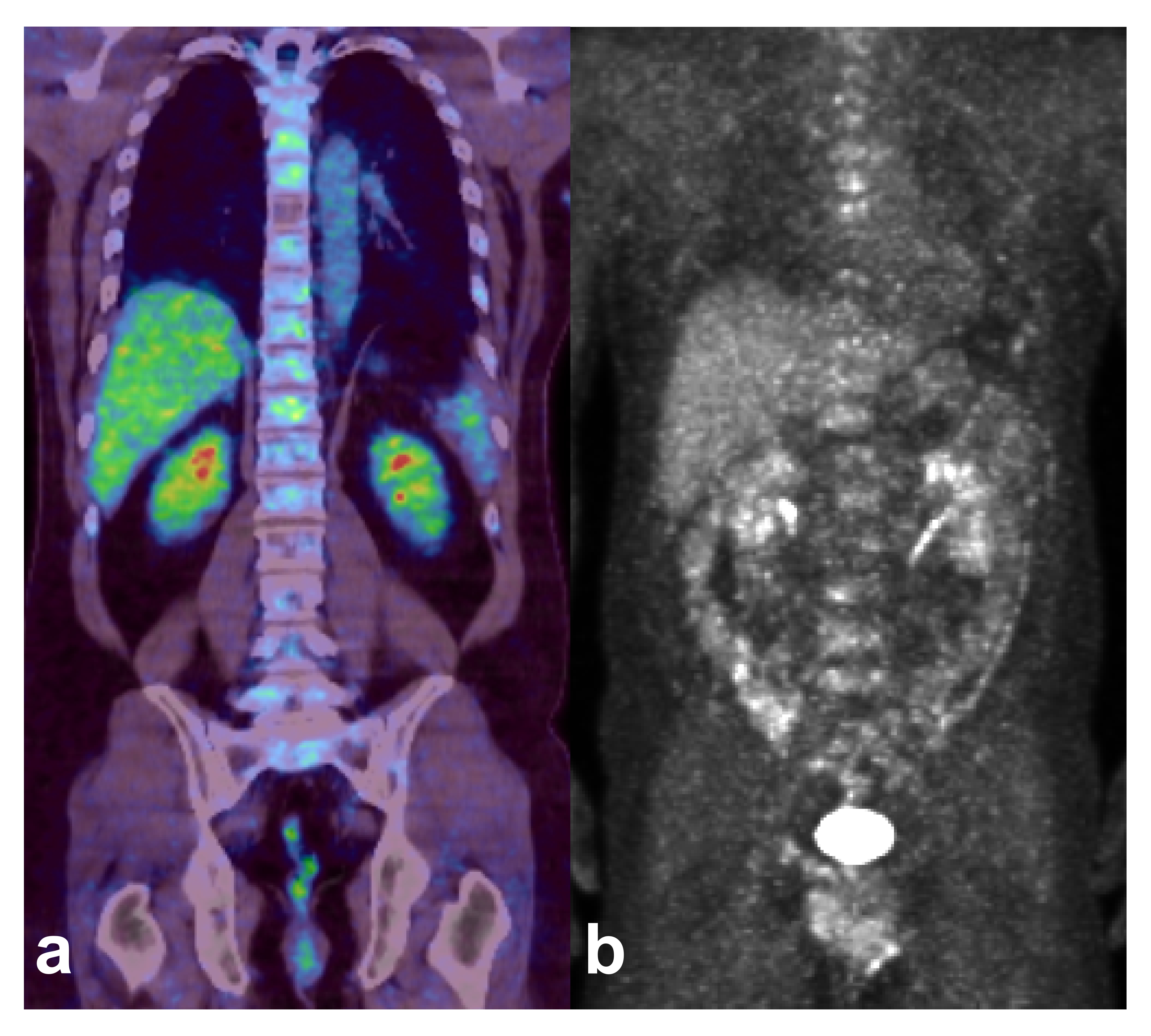

Supplement: Supplementary file 1 — FIGURE S1 Positron emission tomography‐computed tomography. Coronal fused positron emission tomography‐computed tomography (PET‐CT) images were obtained using fluorodeoxyglucose (FDG). Volume control image showing focal FDG uptake, showing localization to the bone marrow (a). The delayed‐phase maximum intensity projection image highlights increased uptake in the bone marrow (b). PET‐CT with FDG uptake images in the bone marrow, raising the possibility of bone metastasis, although the findings were atypical for pancreatic cancer; however, the absence of lytic or sclerotic lesions and the patient's known MDS supported a diagnosis of ineffective hematopoiesis and compensatory marrow hyperplasia rather than metastasis. [file DEO2-6-e70241-s005.jpg]

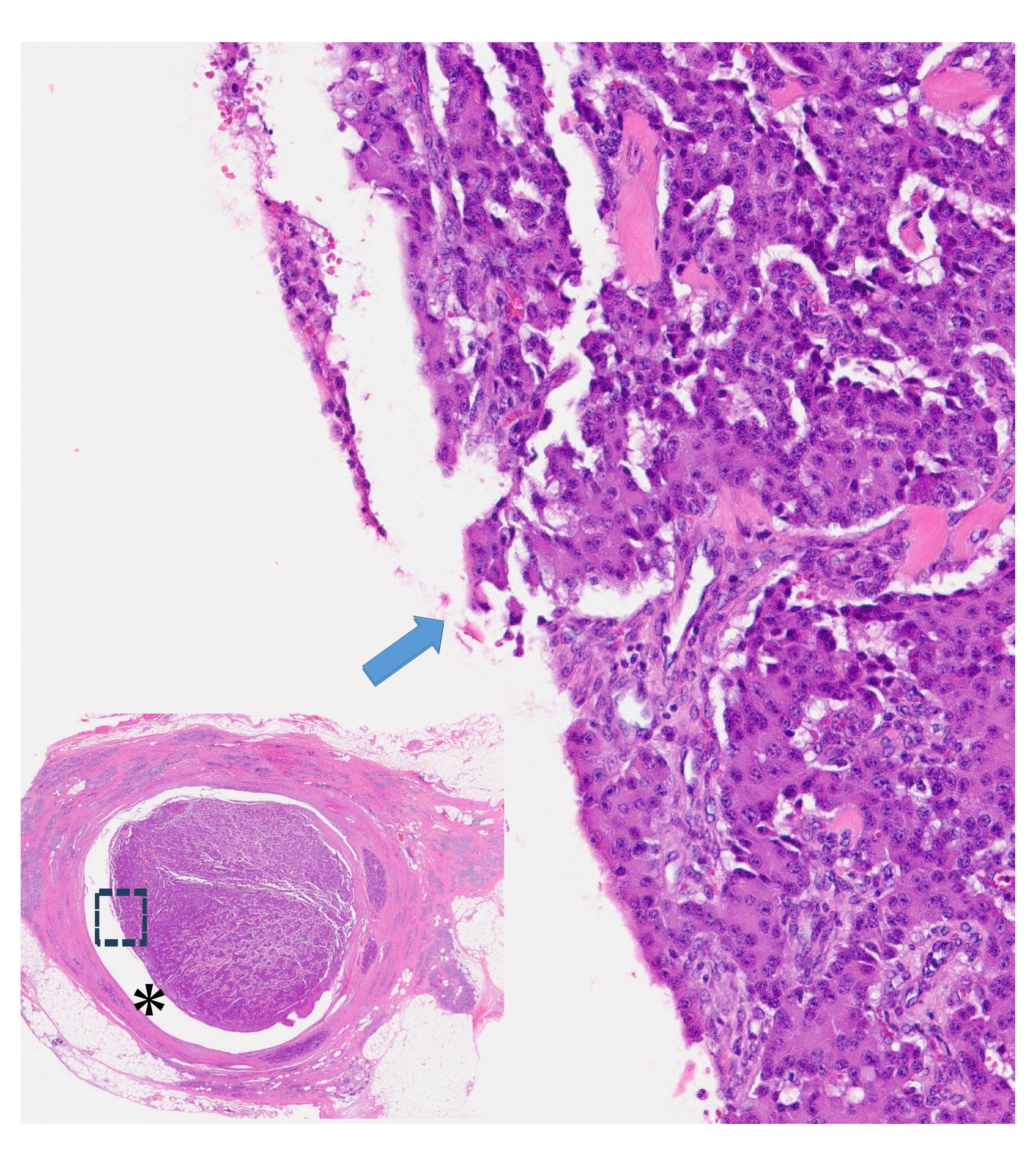

Supplement: Supplementary file 2 — FIGURE S2 Pancreatic neuroendocrine tumor protruding into the main pancreatic duct and tumor cells exposed within the ductal lumen. The pain pancreatic duct lumen (asterisk) and magnified view (box) demonstrate epithelial denudation of the duct wall, with tumor cells (arrow) directly exposed to the ductal lumen. [file DEO2-6-e70241-s004.jpg]

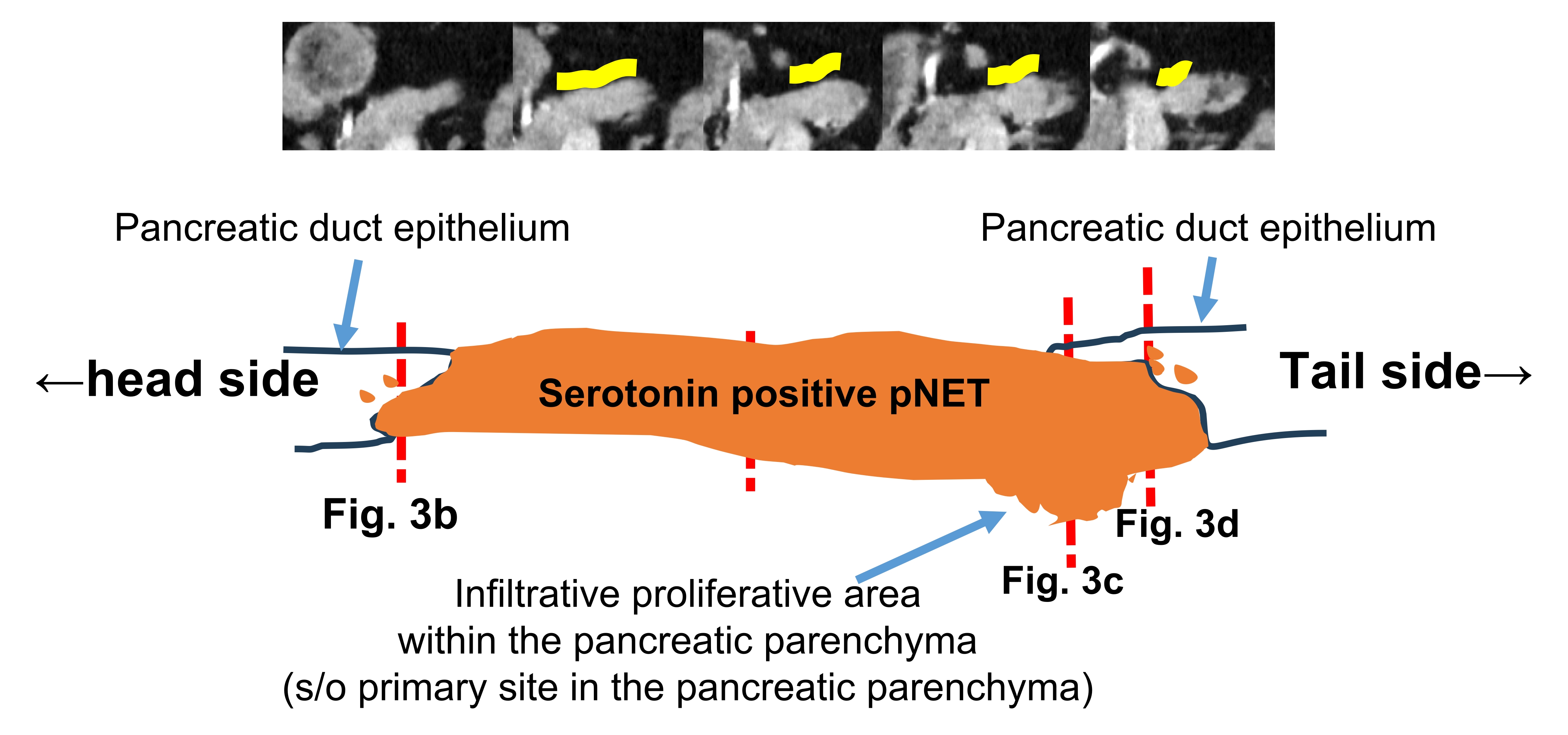

Supplement: Supplementary file 3 — FIGURE S3 Schematic representation of a serotonin‐positive pancreatic neuroendocrine tumor arising from the margin of the main pancreatic duct and protruding into the ductal lumen. Tumor enlargement, mechanical stress, and shear forces between the tumor and the pancreatic parenchyma eventually caused disruption of the fibrous capsule, resulting in exfoliation of tumor cells into the main pancreatic duct. Cytological evaluation was performed using an endoscopic nasobiliary drainage (ENBD) tube. During the first SPACE examination, only class II cytology was obtained, with no definitive tumor cells detected. Although imaging findings showed no apparent differences between the first and second examinations, the second SPACE examination yielded class V cytology. This discrepancy was considered to reflect the progression of capsular rupture due to tumor growth, which facilitated the detachment and shedding of tumor cells into the ductal lumen, allowing their recovery through the ENBD tube. The yellow line delineates the extent of the tumor. [file DEO2-6-e70241-s003.jpg]
