# Supplementary material for: A Case of Pancreatic Neuroendocrine Tumor Growing Intraductal Extension toward the Main Pancreatic Duct Complicated by Thrombocytopenia: Diagnostic Challenges and Management Strategy
Source: DEN Open. 2025 Nov 3;6(1):e70241. doi: 10.1002/deo2.70241 (PMC12582909; doi:10.1002/deo2.70241)
Supplement: Supplementary file 4 — TABLE S1 Differential diagnosis of main pancreatic duct (MPD)–occupying tumors. [file DEO2-6-e70241-s001.docx]

**Table S1. Differential diagnosis of main pancreatic duct–occupying tumors**

Abbreviations: MPD, main pancreatic duct; IPMN, intraductal papillary mucinous neoplasm; IPMC, intraductal papillary mucinous carcinoma; ITPN, intraductal tubulopapillary neoplasm; IOPN, intraductal oncocytic papillary neoplasm; PDAC, pancreatic ductal adenocarcinoma; NET, neuroendocrine tumor; ACC, acinar cell carcinoma.

| **Entity** | **Mechanism of MPD occupation** | **Mucin** | **Imaging clues** |
| --- | --- | --- | --- |
| Main-duct IPMN / IPMC | Papillary, mucin-rich epithelial proliferation fills and dilates the MPD | Positive (abundant) | Diffuse MPD dilatation on MRCP; “fish-mouth” papilla with mucin extrusion; mural nodules on EUS |
| ITPN | Solid intraductal tubulopapillary tumor plugs the MPD (mucin-poor) | Negative / scant | Solid, relatively well-circumscribed intraductal mass; less mucinous signal |
| IOPN | Oncocytic epithelial papillae expand and occupy the duct lumen | Positive | Lobulated cystic–solid lesion with enhancing intracystic nodules |
| PDAC | Invasive carcinoma protrudes into the MPD and obstructs the lumen | Negative | Hypovascular mass with MPD cutoff and upstream dilatation; focal intraductal soft-tissue |
| Pancreatic NET | Polypoid intraductal growth occludes the MPD; may arise from parenchyma and extend into the duct | Negative | Hypervascular mass on arterial phase; duct-filling defect; sometimes pancreatitis |
| Acinar cell carcinoma | Acinar tumor casts the MPD in a “plug/sausage-like” fashion; long-segment intraductal extension; may trigger pancreatitis | Negative | EUS shows an intraductal tumor thrombus with upstream MPD dilatation |

In the differential diagnosis of main pancreatic duct (MPD)-occupying lesions, it is crucial to distinguish intraductal pNET from other intraductal tumors such as IPMN, ITPN, and PDAC. Intraductal or duct-protruding pNETs typically show intense enhancement in the arterial phase owing to their hypervascularity, whereas IPMNs are characterized by mucin production, ductal dilatation, and papillary projections with only mild enhancement. ITPNs present as solid intraductal masses with little or no mucin secretion and demonstrate homogeneous enhancement less intense than pNETs. In contrast, PDAC generally appears hypovascular in the arterial phase, causes ductal stenosis or obstruction, and often shows periductal infiltration rather than a true intraductal growth. These imaging features provide important clues for the preoperative differential diagnosis, although histological confirmation is often required for definitive diagnosis.
